# Teneurin C-terminal associated peptide (TCAP)-1 attenuates the development and expression of naloxone-precipitated morphine withdrawal in male Swiss Webster mice

**DOI:** 10.1007/s00213-024-06582-0

**Published:** 2024-04-17

**Authors:** Lauren E. Mueller, Roseanne S. Wexler, David A. Lovejoy, Robert B. Stein, Andrew M. Slee

**Affiliations:** 1 Protagenic Therapeutics, Inc., New York, NY USA; 2 NeoSome Life Sciences, Lexington, MA USA; 3 Department of Cell and Systems Biology, University of Toronto, Toronto, CA Canada

**Keywords:** Teneurin C-terminal associated peptide-1, Morphine withdrawal, Corticotropin-releasing factor, Corticosterone, Mice

## Abstract

**Rationale:**

Corticotropin-releasing factor (CRF), the apical stress-inducing hormone, exacerbates stress and addictive behaviors. TCAP-1 is a peptide that directly inhibits both CRF-mediated stress and addiction-related behaviors; however, the direct action of TCAP-1 on morphine withdrawal-associated behaviors has not previously been examined.

**Objective:**

To determine whether TCAP-1 administration attenuates behavioral and physiological consequences of morphine withdrawal in mice.

**Methods:**

Mice were administered via subcutaneous route TCAP-1 either before or after initial morphine exposure, after which jumping behavior was quantified to assess the effects of TCAP-1 on naloxone-precipitated morphine withdrawal. As a comparison, mice were treated with nonpeptide CRF_1_ receptor antagonist CP-154,526. In one experiment, plasma corticosterone (CORT) was also measured as a physiological stress indicator.

**Results:**

Pretreatment with TCAP-1 (10–250 nmol/kg) before morphine treatment significantly inhibited the development of naloxone-precipitated withdrawal. TCAP-1 (250–500 nmol/kg) treatment administered after morphine treatment attenuated the behavioral expression of naloxone-precipitated withdrawal. TCAP-1 (250 nmol/kg) treatment during morphine treatment was more effective than the optimal dosing of CP-154,526 (20 mg/kg) at suppressing the behavioral expression of naloxone-precipitated withdrawal, despite similar reduction of withdrawal-induced plasma CORT level increases.

**Conclusions:**

These findings establish TCAP-1 as a potential therapeutic candidate for the prevention and treatment of morphine withdrawal.

## Introduction

Although opioids such as hydrocodone, oxycodone, and tramadol are extremely effective analgesics, their over-prescription fueled the rise of a large drug abuse epidemic due to the rewarding properties they also possess (Meldrum 2020; National Academies of Sciences et al., 2017). Due to public health initiatives, opioid prescription for pain management has been moderated in recent years (Alsultan and Guo 2022; Ball et al. 2021; Dowell et al. 2016; Flanagan et al. 2020), accompanied by a corresponding drop in prescription opioid overdose deaths (NIDA 2023). However, a non-prescription opioid epidemic continues in its wake, with synthetic opioid use now coming to the forefront (Friedman et al. 2022; NIDA 2023). One well-established consequence of opioid use is dependence, which includes a reluctance to discontinue opioids and the presentation of withdrawal symptoms when opioid use is halted. The magnitude of opioid withdrawal typically correlates with the duration and degree of opioid exposure, lasting for days or weeks in long-term heavy opioid users (Dydyk et al. 2023; WHO 2009). However, even short-term opioid exposure can result in acute, albeit less severe, withdrawal, that may nonetheless be sufficient to motivate subsequent use (Abdelhamid et al. 1991; Elhabazi et al. 2012; Gold et al. 1994; Heishman et al. 1989; Kest et al. 2001; Seth et al. 2011; Wilson et al. 2021; Yano and Takemori 1977). In rodents (Kamei et al. 1973; Kest et al. 2001, 2002) and humans (Bickel et al. 1988; Jones et al. 2020), withdrawal from opioids can be precipitated by administration of the opioid antagonist, naloxone. A behavioral hallmark of naloxone-precipitated opioid withdrawal in rodents is the induction of jumping behavior (Kamei et al. 1973; Kest et al. 2001, 2002; Singh et al. 2015; Wei et al. 2023).

Endogenous opioids like endorphins, dynorphins, and enkephalins act as part of the CRF-associated stressor response (Houshyar et al. 2003). Briefly, following a stressful sensory input, an increase in hypothalamic CRF initiates the hypothalamus-pituitary-adrenal (HPA) response, a cascade of events eventually resulting in the release of glucocorticoids from the adrenal cortex. One such glucocorticoid is corticosterone (CORT) in rodents, or cortisol in humans, a mediator of both behavioral and physiological stress responses (Vale et al. 1981). Withdrawal from drugs of abuse increases CRF levels both within the hypothalamus and other brain regions (Buckingham 1982; Funk et al. 2006; George et al. 2007; Merlo Pich et al. 1995; Richter and Weiss 1999; Roberto et al. 2010; Weiss et al. 2001; Zorrilla et al. 2001), indicating that withdrawal is a stressful experience. Accordingly, pre-treatment with CRF receptor antagonists reduce behavioral manifestations of withdrawal (Brugger et al. 1998; Funada et al. 2001; Iredale et al. 2000; Lu et al. 2000; McNally and Akil 2002; Skelton et al. 2007) and CRF_1_ receptor knockout mice demonstrate reduced withdrawal-associated behaviors (Contarino and Papaleo 2005). Together, these findings suggest a role for CRF in mediating withdrawal from drugs of abuse.

Teneurin C-terminal Associated Peptide (TCAP)-1 has been identified as a stress response regulator, likely acting through an HPA-independent CRF-related mechanism (Al Chawaf et al. 2007; Chen et al. 2013; Tan et al. 2009; Wang et al. 2005). In situ hybridization studies showed that TCAP-1 mRNA is highly expressed in brain regions that express CRF receptors and are important for the regulation of stress, such as the hypothalamus, cerebellum, and limbic system (Chen et al. 2013; Wang et al. 2005). Administration of TCAP-1 before CRF decreased CRF-induced c-Fos expression in these regions (Tan et al. 2009), suggesting that TCAP-1 blocks stress-related CRF activity. Using two behavioral assays commonly employed to study anxiolytics, administration of TCAP-1 attenuated the CRF-associated acoustic startle response and increased time spent in the open arm on an elevated plus-maze (Tan et al. 2011; Wang et al. 2005). More relevant to the present study, TCAP-1 has been directly implicated in aspects of substance use disorder (SUD), with repeated treatment attenuating both CRF-induced reinstatement of cocaine seeking and cocaine-sensitized CRF-induced locomotion (Kupferschmidt et al. 2011). These studies establish that TCAP-1 inhibits both CRF stress and addiction-related behaviors, but these attenuating effects on behavior associated with cocaine – a psychostimulant – may not necessarily generalize to other classes of psychoactive drugs, such as opioids. However, because the opioid-associated neurological circuit encompasses both stress- and addiction-related behaviors (Crowley and Kash 2015; Haun et al. 2022; Leconte et al. 2022; Rysztak and Jutkiewicz 2022; Simmons et al. 2020; Tkaczynski et al. 2022; Zhang et al. 2022), and because opioid cessation results in withdrawal which is itself as stressor, it is possible that TCAP-1 could have utility in facilitating opioid abstinence.

Therefore, the effects of TCAP-1 on the behavioral actions of morphine withdrawal were examined, together with a pharmacological comparison of the behavioral and physiological effects of TCAP-1 and nonpeptide CRF_1_ receptor antagonist CP-154,526. First, a naloxone-precipitated morphine withdrawal assay was utilized (Kamei et al. 1973; Kest et al. 2002; Tanganelli et al. 1991), to examine whether administration of TCAP-1 either before or after morphine treatment influenced withdrawal-related jumping behavior. Using a similar design, the efficacy of TCAP-1 and CP-154,526 were compared in attenuating the naloxone-induced jumping response and CORT level increase in morphine-treated mice.

## Methods

### Drugs

Naloxone hydrochloride dihydrate and CP-154,526 [N-butyl-N-ethyl-2,5-dimethyl-7-(2,4,6-trimethylphenyl)pyrrolo[3,2-e]pyrimidin-4-amine] hydrochloride was purchased from Sigma (St. Louis, MO). Morphine sulfate was purchased from Spectrum Chemical (New Brunswick, NJ). Synthetic mouse TCAP-1 (TCAP-1) was synthesized and purchased from Chinese Peptide Company, Incorporated (Hangzhou, China). (+)-MK-801 was obtained through Tocris Bioscience (Bristol, UK). Naloxone and MK-801 were dissolved in 0.9% sterile saline. Morphine hydrochloride was first dissolved in distilled water and then diluted using saline. TCAP-1 was first suspended in distilled water with ammonium hydroxide and vortexed and further diluted using saline. CP-154,526 was dissolved in 10% DMSO and 10% Cremophor EL in saline. Drug doses are expressed in terms of their salt form. Naloxone (2.5 and 10 mg/kg), morphine (32 mg/kg), and MK-801 (0.5 mg/kg) were administered i.p., while TCAP-1 (10–500 nmol/kg) and CP-154,526 (5–50 mg/kg) were given subcutaneously (s.c.).

### Animals

Male Swiss Webster mice (Charles River Laboratories, Kingston, NY) six weeks of age and weighing 20–30 g at experiment onset were group housed on a 12/12-hour light/dark cycle with behavior conducted during the light period. Mice had free access to water and food, except during behavioral test sessions. All housing and experimental conditions complied with Institutional Animal Care and Use Committee guidelines of NeoSome Life Sciences, LLC (Lexington, MA). The number of animals used for each experiment is given in the corresponding figure legend.

### Experimental design

Three studies were conducted to evaluate the effects of TCAP-1 on naloxone-precipitated jumping behavior in morphine-treated mice and the accompanying physiological stress response. First, a dose-range finding study was conducted to establish optimal doses of TCAP-1 to attenuate naloxone-precipitated jumping behavior when TCAP-1 was given prophylactically prior to morphine treatment. Next, the ability of TCAP-1 to attenuate naloxone-precipitated jumping behavior as an intervention was evaluated, after morphine treatment had already commenced. Finally, the ability of TCAP-1 to attenuate both jumping behavior and decrease physiological stress response as reflected by plasma corticosterone concentration was assessed along with a small molecule CRF_1_ receptor antagonist CP-154,526.

### Naloxone-precipitated morphine withdrawal test

**Fig. 1:**
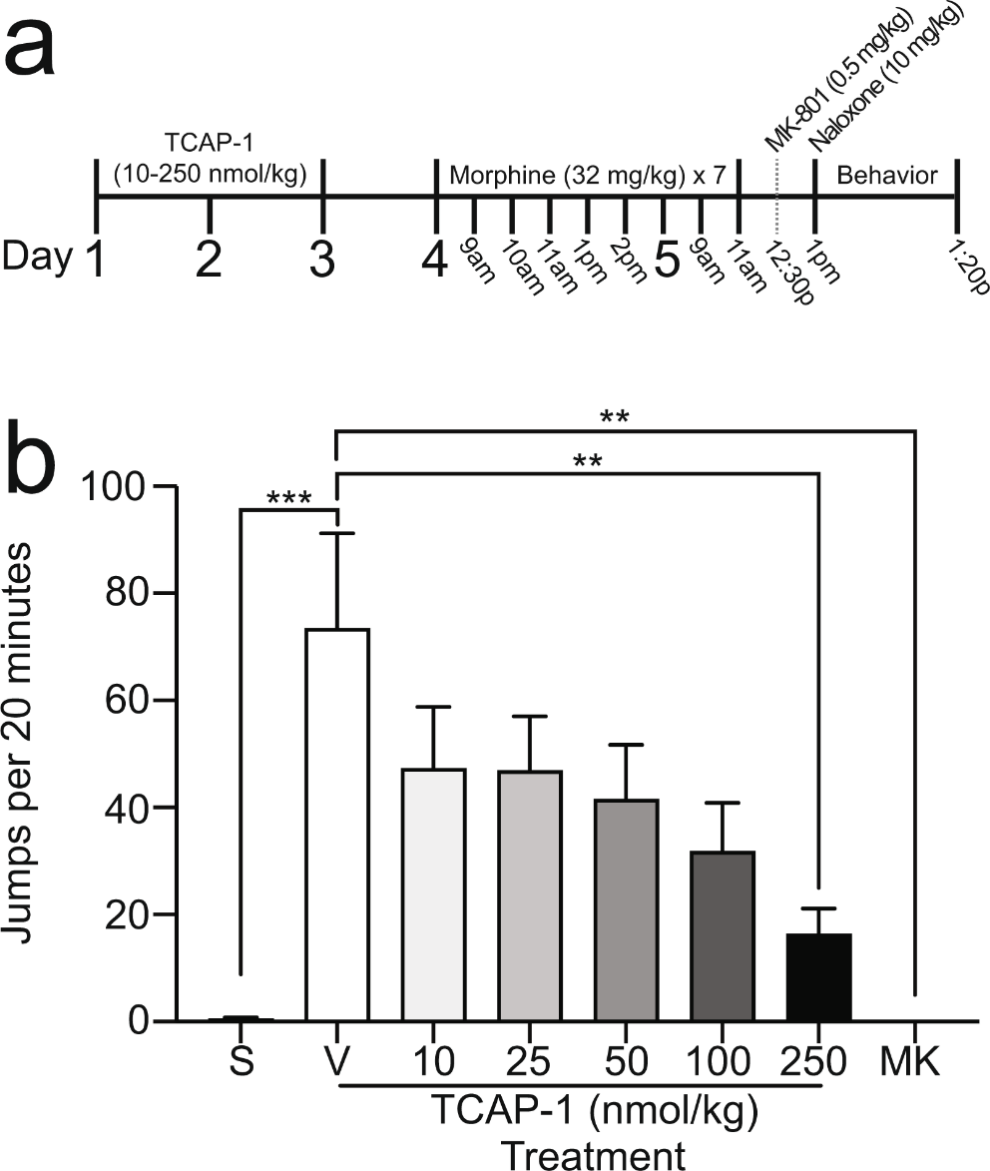
Effects of TCAP-1 pretreatment on naloxone-precipitated morphine withdrawal. **a** Experimental procedure of drug treatment and behavioral measurement. TCAP-1 (10–250 nmol/kg, s.c.) was administered daily for three days prior to morphine treatment. Naloxone (10 mg/kg, i.p.) was administered to induce a behavioral manifestation of withdrawal after the morphine treatment regimen. MK-801 (0.5 mg/kg, i.p.) was administered 30 min prior to naloxone treatment. **b** Number of jumps during a 20 min period after naloxone injection. Data are represented as mean $\pm$ SEM. S = saline, *n* = 9; V = vehicle, *n* = 9; 10–250 nmol/kg TCAP-1, *n* = 9 for each group; MK = MK-801, *n* = 4. *** *P* < 0.001; ** *p* < 0.01

**Fig. 2:**
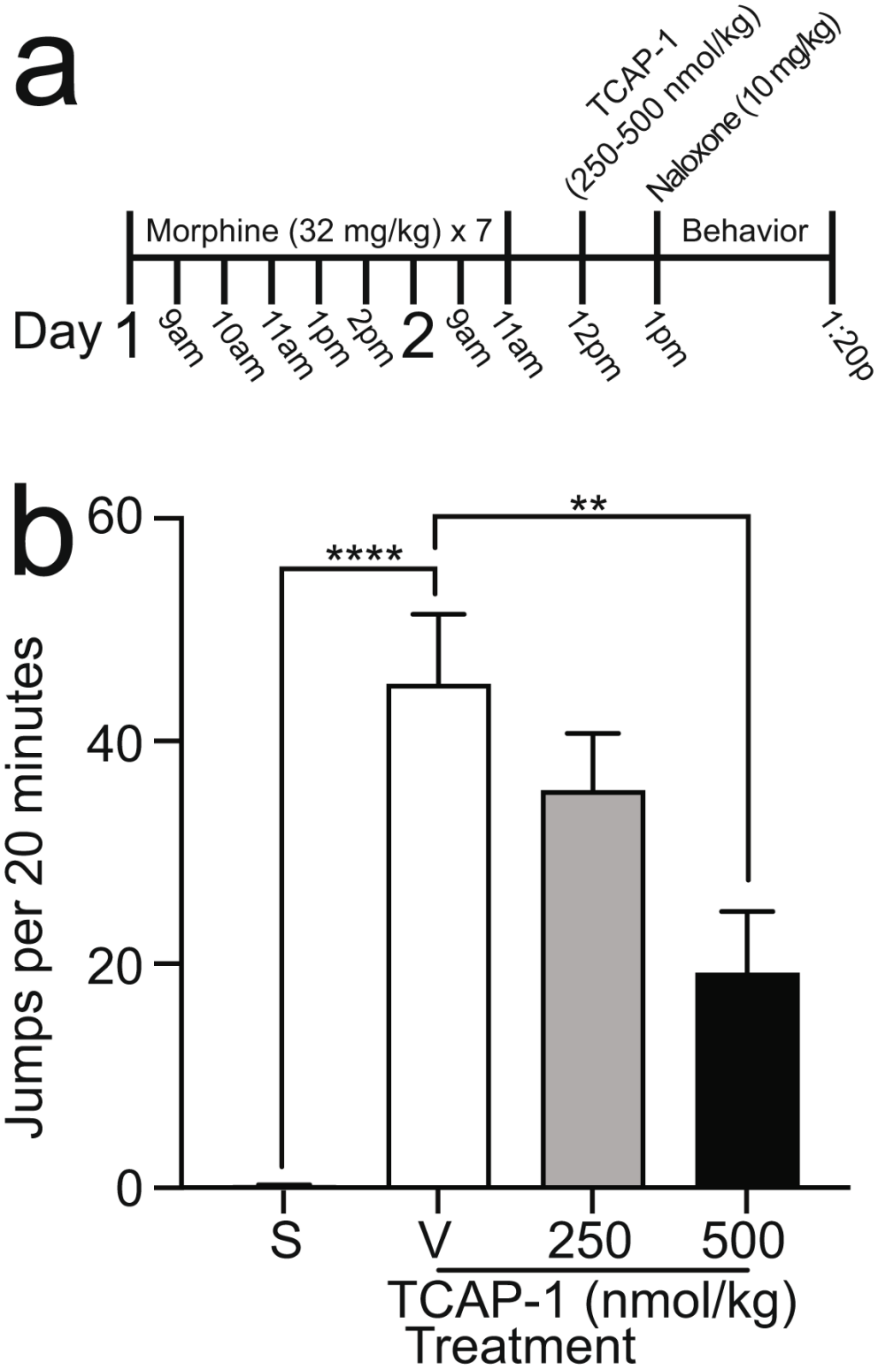
Effects of TCAP-1 treatment post morphine on naloxone-precipitated withdrawal. **a** Experimental procedure of drug treatment and behavioral measurement. TCAP-1 (250–500 nmol/kg, s.c.) was administered once after morphine. Naloxone (10 mg/kg, i.p.) was administered to induce a behavioral manifestation of withdrawal after the morphine treatment regimen. **b** Number of jumps during a 20 min period after naloxone injection. Data are represented as mean $\pm$ SEM. S = saline, *n* = 7; V = vehicle, *n* = 5; 250 nmol/kg TCAP-1, *n* = 6; 500 nmol/kg TCAP-1, *n* = 7. **** *P* < 0.0001; ** *p* < 0.01

**Fig. 3:**
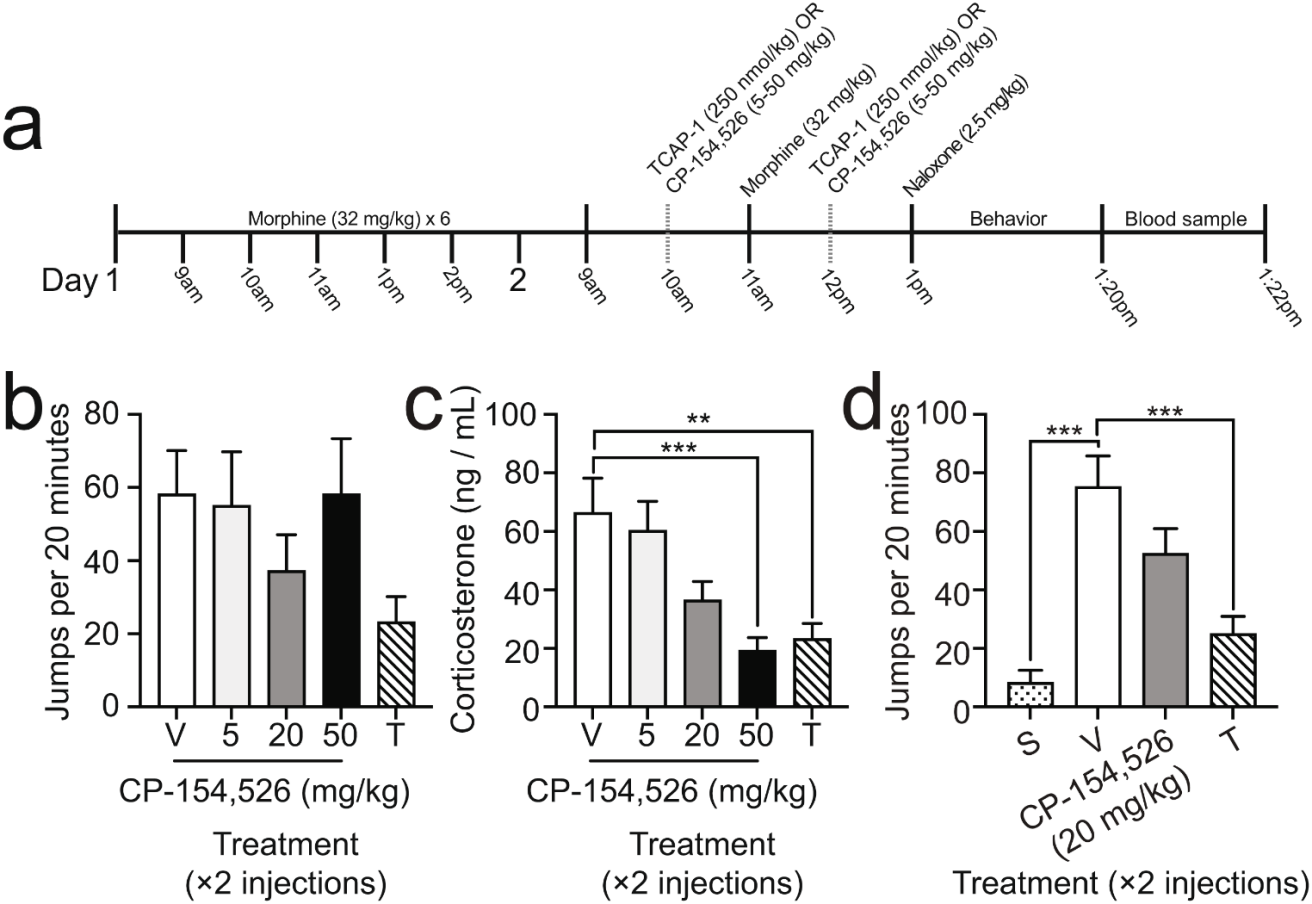
Effects of TCAP-1 or CP-154,526 administration at the end of morphine treatment on naloxone-precipitated withdrawal and plasma corticosterone levels. **a** Experimental procedure of drug treatment and behavioral measurement. TCAP-1 (250 nmol/kg, s.c.) or a dose range of CP-154,526 (5–50 mg/kg, s.c.) was administered in an interleaved fashion one hour before and after the last morphine treatment. Naloxone (2.5 mg/kg, i.p.) was administered to induce a behavioral manifestation of morphine withdrawal. **b** Number of jumps during a 20 min period after naloxone injection. V = vehicle, *n* = 11; 5 and 50 mg/kg CP-154,526, *n* = 11 for each group; 20 mg/kg CP-154,526, *n* = 12; T = 250 nmol/kg TCAP-1, *n* = 12. **c** Plasma corticosterone levels measured from blood taken immediately after jumping. V = vehicle, *n* = 6; 5–20 mg/kg CP-154,526, *n* = 6; 50 mg/kg CP-154,526, *n* = 9; T = 250 nmol/kg TCAP-1, *n* = 6. **d** Comparison of the number of jumps during a 20 min period after naloxone injection between an optimal dose of CP-154,526 and TCAP-1. S = saline, *n* = 4; V = vehicle, *n* = 12; CP-154,526 = 20 mg/kg, *n* = 8; T = 250 nmol/kg TCAP-1, *n* = 12. All data are expressed as mean $\pm$ SEM. *** *P* < 0.001; ** *p* < 0.01

Seven intraperitoneal (i.p.) injections of morphine (32 mg/kg) were spread across two days, with the first three injections occurring in one-hour intervals and the last two injections occurring in two-hour intervals on the first day, and a two-hour interval on the second day. Two hours following the last injection of morphine, mice received one i.p. injection of naloxone (2.5 or 10 mg/kg) and were placed into individual Plexiglas observation cylinders (20 cm H x 12 cm W) where behavior was captured for 20 min. In some experiments, an additional control group of mice were treated with saline instead of morphine and treated with naloxone prior to the observation period (groups denoted ‘S’ in figures). Multiple blinded, independent scorers quantified the number of jumps, defined as the simultaneous removal of all paws, made by each mouse. In the experiment related to Fig. 1, TCAP-1 was administered at doses of 10–250 nmol/kg, and three daily injections were given one day prior to the initiation of morphine. In the experiment related to Fig. 2, TCAP-1 was administered at doses of 250 or 500 nmol/kg one hour following the last morphine injection. In the experiment related to Fig. 3, TCAP-1 was administered at a dose of 250 nmol/kg and CP-154,526 was administered at doses of 5–50 mg/kg; these treatments were administered one hour before and one hour after the last morphine injection.

### Plasma corticosterone assessment

In the experiment related to Fig. 3b, immediately following behavioral sessions, 0.15–0.3 mL of blood was collected and used to detect plasma CORT levels. Briefly, using a 5 mm lancet to prick the submandibular vein, blood was collected into heparin separator mini collect tubes (Greiner Bio-One, Monroe, North Carolina) and placed on ice. Samples were then spun in a refrigerated microcentrifuge at 2,000 x g for eight minutes, after which plasma was extracted. Finally, plasma CORT levels were quantified using a rodent CORT enzyme-linked immunosorbent assay (ELISA, Cayman Chemical Company, Ann Arbor, MI). The process was executed as per the manufacturer’s instruction, with absorbance measured at 420 nm using a microplate reader (Molecular Devices, San Jose, CA).

### Data analysis

All statistical analyses and graphs were performed using Prism 9 (GraphPad Software, San Diego, CA). Values are expressed as mean ± SEM. Statistics were performed on behavioral and ELISA data using one-way analysis of variance (ANOVA) with follow-up Tukey’s tests for multiple comparisons to determine significance. An *a priori* level of significance of *p* < 0.05 was considered significant in these studies.

## Results

### Pretreatment with TCAP-1 attenuates the development of morphine withdrawal

Mice were treated daily with either TCAP-1 across a broad dose range (10–250 nmol/kg) or the control vehicle for three days. On the next day, mice began a morphine treatment assay, receiving a total of seven treatments over days four and five of the paradigm. One group of control mice that did not receive TCAP-1 were given saline injections in place of morphine. Naloxone was administered two hours after the last morphine or saline injection and jumping behavior was quantified immediately after (Fig. 1a). To validate our assay with a positive treatment control group, we included mice treated with MK-801, an N-methyl-D-aspartate (NMDA) receptor antagonist known to reduce the behavioral manifestation of precipitated withdrawal, 30 min prior to naloxone treatment (Cappendijk et al. 1993; Higgins et al. 1992; Koyuncuoğlu et al. 1992; Rasmussen et al. 1991; Trujillo and Akil 1991). There was a significant effect of treatment across groups on morphine-induced jumping behavior (one-way ANOVA, F_7, 59_ = 5.404, *p* < 0.0001, Fig. 1b). These data indicated that, first, the number of jumps in morphine-treated mice was significantly increased compared to saline-treated mice (‘S’ vs. ‘V’, *p* = 0.0001; Tukey’s Test). In contrast, the number of jumps in morphine-treated mice with 250 nmol/kg TCAP-1 treatment was significantly decreased by 78.4% as compared to morphine-treated mice with no TCAP-1 treatment (‘V’ vs. ‘250’, *p* = 0.0045; Tukey’s Test). Finally, as expected, MK-801 completely abolished naloxone-precipitated jumping (‘V’ vs. ‘MK’, *p* = 0.004; Tukey’s Test), supporting the validity of the experimental and scoring protocols. Altogether, these results show that TCAP-1 treatment prior to morphine treatment attenuated the development of naloxone-precipitated jumping.

### Administration of TCAP-1 following morphine treatment attenuates the behavior of the expression of morphine withdrawal

Using the same morphine treatment regimen as the previous experiment, mice were first treated with morphine or saline over two days. One hour following the last injection, mice received one treatment of TCAP-1 or vehicle as the control. Naloxone was administered one hour later, and jumping was quantified (Fig. 2a). There was a significant treatment effect (one-way ANOVA, F_3, 21_ = 17.34, *p* < 0.0001, Fig. 2b). The number of jumps in morphine-treated mice was significantly increased compared to saline-treated mice (‘S’ vs. ‘V’, *p* < 0.0001; Tukey’s Test). Moreover, the number of jumps in morphine-treated mice with 500 nmol/kg TCAP-1 treatment was significantly decreased by 57.5% as compared to morphine-treated mice with no TCAP-1 treatment (‘V’ vs. ‘500’, *p* = 0.0058; Tukey’s Test). These results show that a single administration of TCAP-1 post morphine treatment attenuated the behavioral expression of naloxone-precipitated morphine withdrawal.

### Comparison of the effects of TCAP-1 and CP-154,526 in attenuating the behavioral and physiological expression of morphine withdrawal

We next compared the efficacy of TCAP-1 with a dose range of nonpeptide corticotropin-releasing factor receptor 1 (CRF_1_) antagonist CP-154,526, an agent known to reduce behavioral (Lu et al. 2000) and physiological effects of morphine withdrawal (Navarro-Zaragoza et al. 2021). For this modified treatment design, all mice were treated with morphine over two days, with either TCAP-1 (250 nmol/kg), CP-154,526 (5–50 mg/kg), or vehicle being administered one hour before and one hour after the last morphine injection, to ensure that test compound remained on board before the naloxone-precipitated withdrawal test commenced. Naloxone was administered one hour after the last injection of TCAP-1, CP-154,526, or vehicle, jumping was recorded for 20 min, and blood was collected to measure plasma levels of CORT (Fig. 3a).

There was no significant effect of treatment on naloxone-precipitated jumping, due in part to the biphasic, U-shaped dose-effect produced by CP-154,526 (one-way ANOVA, F_4,52_ = 1.745, *p* = 0.1542, Fig. 3b). Sampled blood was used to measure CORT plasma levels as a biomarker for stress. Unlike the lack of effect observed on behavior, there was a significant treatment effect on CORT plasma levels (one-way ANOVA, F_4,28_ = 7.993, *p* = 0.0002, Fig. 3c). CORT levels in morphine-treated mice with either CP-154,526 (50 mg/kg) or TCAP-1 (250 nmol/kg) treatment were significantly lower by 71.0% and 64.9%, respectively, compared to morphine controls (‘V’ vs. ‘50’, *p* = 0.0008; ‘V’ vs. ‘T’, *p* = 0.0058; Tukey’s Test). Second, concentrations in morphine-treated mice with CP-154,526 (50 mg/kg) treatment were not significantly different from those treated with TCAP-1 (250 nmol/kg; ’CP 50 mg/kg’ vs. ‘T’, *p* = 0.9947; Tukey’s Test), despite the behavioral discrepancy between these treatments noted above.

Though treatments in this cohort did not elicit significant behavioral differences, which was not surprising due to known biphasic curves of CRF_1_ receptor antagonists (Millan et al. 2001; Vasconcelos et al. 2019; Walker et al. 2009a, b), it did establish 20 mg/kg as an optimal dose at which to test the inhibitory effects of CP-154,526 on naloxone-precipitated jumping. Using this established optimal dose of the antagonist we ran a second cohort using the same experimental design to directly compare 20 mg/kg CP-154,526 and 250 nmol/kg TCAP-1 (Fig. 3a). There was a significant effect of treatment on naloxone-precipitated jumping (one-way ANOVA, F_3,32_ = 9.587, *p* = 0.0001, Fig. 3d). The number of jumps in morphine-treated mice was significantly increased compared to saline-treated mice (‘S’ vs. ‘V’, *p* = 0.001; Tukey’s Test). Further, while the number of jumps in morphine-treated mice with 250 nmol/kg TCAP-1 treatment was significantly decreased by 75.1% as compared to morphine-treated mice with no treatment (‘V’ vs. ‘T’, *p* = 0.0005; Tukey’s Test), mice administered with 20 mg/kg CP-154,526 did not demonstrate a significant decrease in jumping behavior compared to controls (‘V’ vs. ‘CP’, *p* = 0.2813; Tukey’s Test). Taken together, these results suggest that TCAP-1 appears to better inhibit the behavioral and physiological expression of naloxone-precipitate morphine withdrawal than CP-154,526.

## Discussion

For the first time the effects of TCAP-1 were investigated on both development and expression of the behavioral manifestation of naloxone-precipitated morphine withdrawal. As demonstrated in previous studies, a single naloxone injection reliably increased jumping behavior in morphine-treated mice. Administration of TCAP-1 either before or after initial morphine treatment attenuated naloxone-induced jumping, indicating the suppression of morphine withdrawal by TCAP-1. Comparison of TCAP-1 with the nonpeptide CRF_1_ receptor antagonist CP-154,526, which was previously shown to reduce the manifestation of withdrawal in the lab setting (Millan et al. 2001; Navarro-Zaragoza et al. 2010), revealed that TCAP-1 better attenuated the behavioral expression compared to the optimal dose of the antagonist. More specifically, whereas CP-154,526 paradoxically achieves maximum attenuating effects on naloxone-precipitated jumping and plasma CORT levels at the different doses of 20 mg/kg and 50 mg/kg, respectively, TCAP-1 achieved significant suppression of both endpoints at the same dose, providing compelling evidence for its role in the mechanisms that manifest opioid withdrawal. It should be noted that only male subjects were included in this study to clearly characterize the effects of TCAP-1 without the potential introduction of estrous cycle-related plasma CORT variability previously observed (Nichols and Chevins 1981). Moreover, male and female rodents exhibit developmental differences in opioid withdrawal-induced behavioral responses, which would have further complicated the characterization of TCAP-1 (Bravo et al. 2020). Nonetheless, future studies that include female subjects are warranted to more accurately characterize the broader potential clinical utility of TCAP-1.

This study supports literature implicating a role for TCAP-1 in aspects of the addiction cycle, specifically, opioid-associated withdrawal, functioning through a CRF-related mechanism for attenuating stress. The finding that the TCAP-1 peptide has profound effects on an evolutionarily novel phenomenon such as withdrawal from drugs of abuse is striking, and likely indicates that the primary influence of TCAP-1 is to reduce withdrawal-associated stress to ameliorate withdrawal. Previous work found that following cocaine self-administration, chronic but not acute pretreatment with TCAP-1 blocked both CRF-induced reinstatement and cocaine-sensitized locomotor stimulation by CRF in rats (Kupferschmidt et al. 2011). In another study, rats underwent either short- or long-access cocaine self-administration and the effect of TCAP-1 on CRF-mediated drug reinstatement was examined. Consistent with the previous findings, the study found that rats treated with TCAP-1 after self-administration but before reinstatement demonstrated less CRF-induced lever pressing compared to controls, suggesting that TCAP-1 suppressed CRF-induced cocaine seeking (Erb et al. 2014). Importantly, these findings did not necessarily predict the results of the current study, since the neural underpinnings of addiction of psychostimulants like cocaine are different from those from other classes of drugs, such as opioids in this case. Chronic TCAP-1 pretreatment has also been shown to attenuate other aspects of neuropsychiatric disorders including stress and anxiety. Chronic TCAP-1 pretreatment decreased acoustic startle responses (Wang et al. 2005) and attenuated CRF-induced anxiety-related behavior in the elevated plus maze and open field tests (Al Chawaf et al. 2007). Therefore, one key finding of the present study is that TCAP-1 was effective in attenuating naloxone-precipitated withdrawal-associated behavior either when given prophylactically (i.e., before morphine treatment began), or as an intervention (i.e., after morphine treatment had initiated but prior to precipitated withdrawal). There is great clinical benefit to treatment which can be given as an intervention, since only in certain scenarios (e.g., planned surgeries) will opioid use be predictable, and a therapeutic drug be able to be administered beforehand.

Though morphine is an effective drug for pain management, its use can lead to physiological dependence, after which discontinuation of the drug elicits symptoms of withdrawal syndrome including anxiety, muscle pain, insomnia, vomiting, and sweating. These unpleasant symptoms can drive individuals to continue using morphine via negative reinforcement, potentially leading to drug misuse and abuse. To mitigate morphine withdrawal, TCAP-1 could be administered at the end of morphine treatment to suppress morphine withdrawal. Unlike current treatments for opioid withdrawal including methadone, buprenorphine, and clonidine that also have a high abuse potential, preliminary studies with TCAP-1 suggest that it is not reinforcing and has low abuse potential (unpublished data).

In addition to the aforementioned studies that examined the effects of TCAP-1 on behavior, studies using CRF antagonists have found that these treatments influence stress- and anxiety-related behavior, which provides a strong rationale for the continued investigation of TCAP-1 as a therapeutic for these indications. Most relevant to our current work, treatment with a variety of CRF antagonists have been shown to attenuate behavioral manifestations of withdrawal from drugs of abuse (Roberto et al. 2017). Specifically, pretreatment with the nonpeptide CRF_1_ receptor antagonist CP-154,526 was previously shown to reduce jumping, teeth chattering, writhing, shaking, and weight loss induced by naloxone injection in morphine treated subjects (Almela et al. 2012; Lu et al. 2000). While these studies found that CP-154,526 was able to attenuate morphine withdrawal-induced jumping, these studies were different from the present study in that they used rats as opposed to mice, and used longer morphine treatment regimens to induce dependence (Iredale et al. 2000; Lu et al. 2000). Therefore, the weaker attenuating effect of CP-154,526 in the present study may result from a species difference or the extent of opioid withdrawal generated over the two days of morphine treatment. Treatment with CP-154,526 has also been shown to attenuate anxiety- and depression-related behaviors (Kehne and Cain 2010), with chronic treatment decreasing immobility time in a forced swim test (Overstreet et al. 2004), and acute treatment increasing the amount of time spent in open arms on an elevated plus maze (Griebel et al. 1998; Lundkvist et al. 1996) and decreasing separation-induced vocalizations in pups (Kehne and Cain 2010). However, despite a vast amount of preclinical data suggesting the utility of CRF antagonists to improve behaviors associated with stress and neuropsychiatric disorders at large, many were translational failures in the clinic due to factors such as poor drug safety, efficacy, and specificity, short duration of preclinical screens, and effects on other components of the CRF system that maintain normal physiology (Shaham and de Wit 2016; Spierling and Zorrilla 2017). The paradoxical biphasic dose-response curve elicited with CP-154,526 treatment found in the present study and other antagonists in previous studies may reflect some of these issues (Millan et al. 2001; Walker et al. 2009a, b).

Specifically, in one human trial it was shown that despite receptor antagonism made evident by decreased cortisol levels, a CRF_1_ receptor antagonist had no behavioral effect on major depressive disorder (Binneman et al. 2008). An additional cross-species study found that while a CRF_1_ receptor antagonist blocked HPA axis activation in rats and reduced cortisol levels in anxious alcohol-dependent women, the ratings for alcohol craving in the women were unaffected (Schwandt et al. 2016). The reasons for this dissociation between efficacy in attenuating circulating stress hormones and lack of efficacy in behavior are still uncertain but may be explained by the theory that CRF_1_ receptors in extrahypothalamic sites of action better predict behavioral efficacy. Several studies have implicated areas such as the amygdala as a critical site of action for the CRF_1_ receptor on behavior (Zorrilla et al. 2014), and sufficient CRF_1_ receptor antagonism in this area perhaps does not occur at the same systemic doses as those that effectively suppress circulating CORT. In addition, it is possible that at the doses of CRF_1_ receptor antagonists that effectively suppress CORT, poor selectivity, which may cause these antagonists to engage other receptors, or poor distribution, which may prevent these antagonists from blocking CRF in important target structures (e.g., the amygdala), can explain this lack of efficacy on behavior.

Accordingly, the reasons why TCAP-1 appears to better impact both the HPA axis response and provide behavioral efficacy at a comparable dose are likely complex. One reason may be that whereas CRF_1_ receptor antagonists like CP-154,526 target the receptor directly, TCAP-1 produces a similar net result to inhibit this pathway but via a different mechanism. Some currently documented mechanisms by which TCAP-1 functions include modulating synaptic plasticity and cytoskeletal dynamics via binding to the G-protein coupled receptor latrophilin (Husić et al. 2019; Woelfle et al. 2015), and by modulating intracellular calcium levels via actions on both the mitochondria and plasma membranes (Hogg et al. 2022). Moreover, some of these functions have been shown to attenuate CRF-induced cellular effects (Hogg et al. 2022). Thus, it is possible that this alternative mechanism for attenuating the downstream mechanisms of CRF_1_ receptor activation can account for the better matching behavioral and physiological endpoints determined by the current study. Furthermore, future pharmacokinetic/pharmacodynamic and selectivity data for TCAP-1 may further support its advantage over CRF_1_ receptor antagonists, either due to enhanced selectivity for the CRF_1_ receptor pathway, or better distribution to central sites of action important for behavior, such as the amygdala. The continued investigation of TCAP-1 in drug abuse contexts is warranted and may provide better clinical efficacy in the CRF-targeted class of treatments than direct CRF1 receptor antagonists.

Lastly, it is worthwhile to briefly note that the dosing regimen for naloxone was slightly altered across studies. The first two experiments were carried out using a naloxone dose of 10 mg/kg established in initial naloxone-precipitated morphine withdrawal studies to induce jumping in mice (Siegel et al. 1975). However, since more recent work has shown in fact more robust jumping behavior elicited at lower doses of naloxone, the naloxone dose for later studies was adjusted to 2.5 mg/kg to increase the magnitude of the jumping response to thus better compare efficacy between TCAP-1 and CP-154,526 (el-Kadi and Sharif 1994). Moreover, while we did not formally test the potential locomotor-suppressing effects of TCAP-1 in this study, which may have had a confounding effect on jumping behavior, several studies have demonstrated that either centrally or systemically administered TCAP-1 does not suppress general motor behavior (Al Chawaf et al. 2007; Kupferschmidt et al. 2011). It was also observed in the video recordings that TCAP-1-treated mice were able to move freely around the test cylinder, indicating no reduction of locomotor activity, in contrast to the MK-801-treated mice, which demonstrated a more generalized suppression of locomotion. Therefore, it is unlikely that the results of the current study are attributable to general motor suppression or sedation. Finally, when evaluating novel treatments for withdrawal in animals, it is important to not only consider their effects on stereotyped motor actions and autonomic responses as we have done in the present study, but also on measures of the withdrawal-associated negative affective state anhedonia, anxiety, and avoidance, which were not examined here. As such, while the current data suggest that TCAP-1 suppresses the positive symptoms associated withdrawal, further work will be needed to indicate whether negative affective symptoms may be expected clinically.

In summary, this study found that TCAP-1 treatment significantly attenuated both the development and expression of the behavioral manifestation of withdrawal in morphine-treated mice. Furthermore, TCAP-1 administration at the end of morphine treatment was better able to decrease the jumping behavior compared to nonpeptide CRF_1_ receptor antagonist CP-154,52*6*. Continued investigation of the function and utility of TCAP-1 in the context of withdrawal and other stress-related neuropsychiatric disorders will potentially lead to the development of new and better therapies in the clinic.
